# Mean Platelet Volume Is a Poor Prognostic Factor in Patients Undergoing Liver Transplantation for Hepatocellular Carcinoma

**DOI:** 10.5152/tjg.2025.24540

**Published:** 2025-01-06

**Authors:** Mustafa Şentürk, Volkan İnce, Orhan Üreyen, Kemal Eyvaz, Burak Işık, Brian I Carr, Sezai Yılmaz

**Affiliations:** 1 Department of Surgery, Liver Transplant Institute, İnonu University, Malatya, Türkiye; 2 Department of General Surgery, İzmir Bozyaka Training and Research Hospital, Health Sciences University, Izmir, Türkiye; 3 Department of General Surgery, University of Health Sciences, Antalya Education and Research Hospital, Muratpaşa, Antalya, Türkiye

**Keywords:** MPV, hepatocellular cancer, transplantation

## Abstract

**Background/Aims:**

Mean platelet volume (MPV) reflects platelet activation. Platelets have an important role in tumor progression and metastasis. In this study, we wanted to investigate the effect of MPV on survival in patients undergoing liver transplantation (LT) for hepatocellular carcinoma (HCC).

**Materials and Methods:**

All data of 376 patients who underwent LT and pathologically diagnosed with HCC were analyzed. By determining the cut-off of MPV (10.2 fL), 2 groups with high and low MPV were formed. The groups were compared within themselves. Factors affecting survival were determined by univariate and multivariate analysis.

**Results:**

When the groups were compared, patients with low MPV had significantly higher platelet counts, larger tumor sizes, lower BMI, and higher recurrence rates. In multivariate analysis, GGT >104, AFP >200 μg/L, largest tumor diameter >5 cm, and lower MPV were found to be independent risk factors that affected the prognosis. Tumor-free survival was significantly worse in the lower MPV group (*P* = .002).

**Conclusion:**

Pre-transplant low MPV may be useful in predicting poor prognosis and a high rate of tumor recurrence in patients with HCC after liver transplantation.

**Main Points**

- Platelets have an important role in tumor progression and metastasis.
- Mean platelet volume (MPV) reflects platelet activation.
- Pre-transplant low MPV may be useful in predicting poor prognosis and a high rate of tumor recurrence in patients with HCC after liver transplantation.

## Introduction

Hepatocellular carcinoma (HCC) represents the most prevalent form of primary liver cancer. On a global scale, it is the sixth most common cancer and the third leading cause of cancer-related mortality.^1^ Liver transplantation (LT) is regarded as the most efficacious therapeutic option for HCC.^2^ Following transplantation, over 80% of HCC patients experience a 5-year recurrence-free survival. Nevertheless, recurrence is observed in approximately 20% of patients following LT.^3^ Recently, various inflammatory markers such as neutrophil-to-lymphocyte ratio (NLR), platelet to lymphocyte ratio (PLR), C-reactive protein (CRP), lymphocyte-to-monocyte ratio, and mean platelet volume (MPV) have been employed to assess immune responses in several diseases, including cancer.^4^ One recent study highlighted the crucial role of platelets in facilitating cancer metastasis. Tumor cells activate platelets, which then promote angiogenesis, tumor proliferation, and dissemination. Mean platelet volume is the clinical marker that reflects platelet activation and size.^5^ Previous studies have shown that colorectal cancer patients with higher MPV levels tend to have poorer survival rates compared to those with normal MPV levels.^6^ Survival in HCC patients undergoing LT is associated with a decreased preoperative MPV.^4^ Additionally, MPV has been utilized to predict the occurrence of isolated bone metastases in breast cancer patients.^7^ In gastric cancer, elevated preoperative MPV has been linked to a reduction in overall survival (OS) and disease-free survival (DFS).^8^ In non-small cell lung cancer, a low mean platelet volume has been linked to a poor prognosis after curative surgery.^9^ Mean platelet volume levels have been shown to vary across different cancers, including HCC, breast cancer, gastric cancer, and lung cancer.^4-9^

Mean platelet volume is a routinely measured parameter in hemogram tests. Research investigating the relationship between MPV and the outcomes of patients receiving LT for HCC is limited. In this study, we investigated how MPV levels influence survival in patients who received liver transplants as a treatment for HCC.

## Materials and Methods

### Patient Cohort

Patient cohort for this study consisted of individuals who received living donor liver transplantation for HCC at the İnonu University Liver Transplantation Institute from March 2006 to November 2021. All patients provided informed consent, and data were gathered retrospectively from a prospectively maintained database (retrospective cohort). Ethical approval for the study was obtained from the İnonu University Liver Transplantation Institute (approval number: 2022/3777, date: 06/09/2022).Informed consent forms were obtained from the patients. The medical records included data on demographic and clinical characteristics. Pathological assessments were based on the postoperative pathology reports of the patients. Biochemical parameters examined included albumin, total bilirubin, international normalized ratio (INR), AST, ALT, serum creatinine (Scr), platelet count (Plt), PLR, NLR, alpha-fetoprotein (AFP), and gamma-glutamyl transferase (GGT). Clinical and histopathological evaluation covered variables such as CHILD, MELD scores, histological differentiation, venous invasion, tumor count (>3), largest tumor diameter (>5 cm), Milan criteria, Malatya criteria, Extended Malatya criteria, recurrence, etiology, body surface area (BSA), and body mass index (BMI). Patients were followed up in accordance with National Comprehensive Cancer Network guidelines. The main outcome measure was OS, which was defined as the duration from operation to death, irrespective of the underlying reason. Analyzed were responses from 396 patients who had LT for pathologically proven HCC. Following the exclusion of cadaveric transplants, prior liver transplant recipients, and patients who were lost to follow-up, the final analysis comprised 376 patients.

### Characteristic Selection

The area under the curve (AUC) and the cut-off value for preoperative MPV were determined using receiver operating characteristic (ROC) analysis, with the cut-off set at 10.2 fL. Patients with MPV below the cut-off were classified as having low MPV, while those with values above the cut-off were classified as having high MPV, creating two distinct groups. In this study, AFP and GGT cut-off values of 200 ng/mL and 104 U/L, respectively, were used based on previous research.^2,10^

### Statistical Analysis

Data were presented as median (min-max) and frequency values. The AUC and cut-off value for preoperative MPV were identified using ROC analysis. Differences in clinicopathological factors between the low and high MPV groups were assessed using independent sample *t*-tests, Fisher’s exact test, and Pearson’s *χ*^2^ test. The Kaplan–Meier method was initially employed to identify survival risk factors for HCC. Factors identified were further analyzed in both univariate and multivariate analyses using the Cox regression method. All statistical analyses were performed using SPSS version 22.0 (IBM SPSS Corp.; Armonk, NY, USA), with a type I error threshold set at 0.05 for interpreting the statistical hypothesis tests.

## Results

### Recipient Demographics

The study included a total of 376 liver transplant recipients. Of these, 52 patients (13.8%) were female, while 340 (86.2%) were male. The median age was 56 years, ranging from 5 to 69 years. Preoperative cut-off values for various clinical parameters were established as follows: MPV at 10.30 fL, albumin at 2.9 g/dL, INR at 1.32, total bilirubin at 1.8 mg/dL, and Scr at 0.78 mg/dL. The Kaplan–Meier method was employed to analyze the impact of these factors on tumor-free survival (TFS). Among the cohort, 98 patients had a largest tumor diameter greater than 5 cm, 175 exhibited venous invasion (either micro or macro), and 83 had more than 3 tumors (Table 1). A comparative analysis between low and high MPV groups showed that patients in the low MPV group had a significantly higher platelet counts, larger tumor size, lower BMI, higher recurrence rates, and lower compliance with Milan criteria.

### Survival

**Figure 1.**
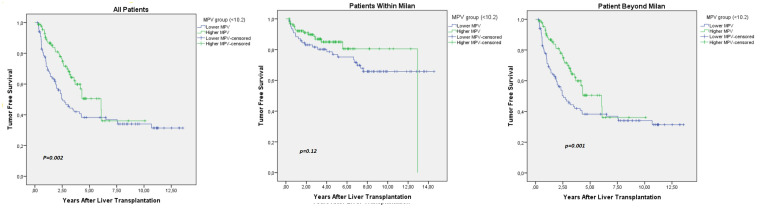
Survival effect of lower and higher MPV groups.

The median follow-up period for the patients was 9 years, ranging between 8.2 and 9.7 years. Tumor-free survival rates at 1, 5, and 10 years were 89.7%, 80.2%, and 70.3%, respectively. A total of 75 patients succumbed to tumor recurrence post-transplantation. To evaluate the influence of MPV on OS after LT, a univariate analysis was conducted. Several factors were identified as significant risk factors for poor prognosis in terms of both TFS and OS, including AFP 200 μg/L, venous invasion, more than 3 tumors, largest tumor diameter >5 cm, MPV <10.2 fL, and GGT >104 U/L. In the multivariate analysis, GGT >104 U/L, AFP >200 μg/L, largest tumor diameter >5 cm, and low MPV emerged as independent risk factors for worse TFS and OS (Table 2). The lower MPV group demonstrated significantly poorer TFS (*P* = .002), as illustrated in Figure 1.

## Discussion

Despite considerable advancements in diagnosing and treating HCC, the overall 5-year survival rate remains disappointingly low. Therefore, identifying new and reliable tumor markers is crucial to improving cancer management and outcomes.^11^ This study’s primary finding is that low MPV is strongly correlated with poorer tumor characteristics and worse prognosis in HCC patients following LT.

Recent research highlights the significant role of platelets in cancer progression and metastasis.^12^ Platelet activation has been shown to enhance cancer cell proliferation, invasion, and angiogenesis. However, the total platelet count can fluctuate depending on platelet consumption and production. The body’s compensatory mechanisms can obscure hypercoagulable states and proinflammatory tumor phenotypes, even when the platelet count appears normal.^13^ Mean platelet volume serves as a reliable indicator of platelet activation. For instance, in a study involving 496 patients with non-small cell lung cancer, elevated MPV was associated with poor OS, suggesting its role as a prognostic biomarker.^14^ Similarly, in 264 metastatic colorectal cancer patients undergoing first-line chemotherapy, those with low MPV had worse survival outcomes.^15^ Another study found that low MPV before chemotherapy was an independent predictor of poor prognosis in patients with extensive large B-cell lymphoma.^16^ Additionally, high MPV was linked to poorer prognosis in a study of 168 patients with resectable gastric cancer.^8^ Collectively, these findings demonstrate that both high and low MPV levels significantly impact survival across various tumor types. Specifically, in an analysis of 304 HCC patients post-LT, low MPV was identified as a poor prognostic factor.^9^ Our study yielded similar results, confirming these associations. Multivariate Cox regression analysis identified tumor size >5 cm, AFP > 200 μg/L, GGT >104 µ/L, and MPV <10.2 fL as independent indicators of poor prognosis in HCC patients.

Larger platelets, as well as megakaryocytes, are found in the human spleen and bone marrow.^17^ These large platelets are more functionally active and contain a greater concentration of intracellular thromboxane A2 granules and procoagulant surface proteins.^18^ Consequently, elevated MPV is commonly associated with thrombotic conditions and is used as an indicator of a prothrombotic state.^19^ Conversely, low MPV is observed in advanced tumors and inflammatory diseases and may indicate platelet granule depletion.^20^ Mean platelet volume is routinely measured in complete blood count tests and provides insight into platelet size and activity. Numerous studies support the notion that MPV could serve as a biomarker for predicting survival in patients with various malignancies.^4^ In this context, low MPV reflects degranulated platelets that have already released their tumor-promoting cytokines, linking it to poorer prognosis.^21^ The limitations of our study include its retrospective nature, the relatively small patient sample, and its focus on a single-center cohort of HCC patients undergoing LT.

Reduced MPV could act as a prognostic indicator for unfavorable outcomes and a higher risk of tumor recurrence in HCC patients after LT.

## Figures and Tables

**Table 1.** Characteristics and Comparison Between Patients with Lower MPV (<10.2 fL) and Higher MPV (>10.2 fL)

Variables	Lower MPV	Higher MPV	*P*
Sex (male)			.55
Male	153 (85.5%)	171 (86.8%)	
Female	26 (14.5%)	26 (13.2%)	
Age (years)	54.1 ± 12.2	50.8 ± 14.3	.08
Albumin (g/L)	3 ± 0.66	2.91 ± 0.71	.216
Child			.2
A	63 (35.2%)	66 (33.5%)	
B	70 (39.1%)	99 (47.2%)	
C	46 (25.7%)	38 (19.3%)	
MELD (<15)	112 (62.6%)	119 (60.4%)	.37
Total bilirubin (μmol/L)	3.29 ± 5.51	3.31 ± 4.43	.984
INR	1.4 ± 0.4	1.4±0.4	.594
AST	97.1 ± 119.6	127.1 ± 555.2	.48
ALT	79.7 ± 177.8	78.2 ± 253.1	.94
Scr (μmol/L)	0.8 ± 0.19	0.91 ± 0.96	.160
**Plt count**	**134.88 ± 94.49**	**101.49 ± 58.8**	**<.001**
**PLR**	**121.9 ± 96.1**	**81.2 ± 43.4**	**<.001**
**NLR**	**4.1 ± 4.7**	**3.1 ± 2.7**	**.007**
AFP (μg/L)	355.36 ± 1814.88	413.17 ± 1755.66	.756
GGT (U/L)	114.3 ± 143.1	100.1 ± 81.9	.235
Differentiation			.84
Well	71 (39.7%)	84 (42.6%)	
Moderate	77 (43%)	80 (40.6%)	
Poor	31(17.3%)	33 (16.8%)	
Venous invasion	87 (50.3%)	88 (43.3%)	.107
Tumor number >3	40 (23.8%)	43 (21.4%)	.330
**Within Milan**	**78 (43.6%)**	**108 (54.8%)**	**.01**
Within Malatya	105 (58.7%)	125 (63.5%)	.19
Within Ext Malatya	119 (66.5%)	135 (68.5%)	.377
**The largest tumor diameter >5 cm**	**60 (34.7%)**	**38 (18.7%)**	**.001**
**Recurrence**	**43 (24.9%)**	**32 (15.8%)**	**.01**
Etiology (viral)	146 (84.4%)	159 (78.3%)	.085
BSA	**1.8** **± 0.2**	**1.9** **± 0.2**	.08
**BMI**	**25.46 ± 4.3**	**26.69 ± 4.4**	**.008**

LT: Liver transplantation, MPV: Mean platelet volume, BMI: Body mass index, Scr: Serum creatinine, PLR: Platelet to lymphocyte ratio, NLR: Neutrophile to lymphocyte ratio, BSA: Bdy surface area.

**Table 2.** Univariate and Multivariate Analysis of Variables Influencing Overall Survival After LT

	Univariate	*P*	Multivariate	*P*
	HR (95% CI)		HR (95% CI)	
Sex (male)	0.99 (0.61-1.6)	.98		
Age (>56 yr)	1.08 (0.74-1.56)	.68		
Albumin (>37.49 g/L)	1.04 (0.81-1.46)	.81		
Total bilirubin (>1.8 μmol/L)	1.1 (0.78-1.55)	.57		
INR (>1.38)	1 (0.65-1.54)	.98		
Scr (>0.78 μmol/L)	1.17 (0.83-1.64)	.35		
Plt count (>95.5)	1.25 (0.89-1.76)	.19		
**Venous invasion**	**2.45 (1.72-3.49)**	**<.001**	1.15 (0.74-1.79)	.52
**Tumor number >3**	**1.79 (1.23-2.62)**	**.002**	1.4 (0.94-2.1)	.09
**The largest tumor diameter >5 cm**	**3.63 (2.58-5.12)**	**<.001**	**3.02 (2-4.55)**	**<.001**
**AFP (>200 μg/L)**	**1.97 (1.35-2.88)**	**<.001**	**1.71 (1.14-2.57)**	**.009**
**GGT (>104 U/L)**	**2.07 (1.47-2.91)**	**<.001**	**1.77 (1.22-2.56)**	**<.003**
**MPV (<10.2 fL)**	**0.58 (0.4-0.84)**	**.004**	**0.64 (0.43-0.93)**	**.02**
Etiology (viral)	0.95 (0.82-1.19)	.95		
BMI <25.7	0.83 (0.59-1.18)	.31		

MPV: Mean platelet volume, NLR: Neutrophil to lymphocyte ratio, PLR: Platelet to lymphocyte ratio, Scr: Serum creatinine, BSA: Body surface area, Ext: Extended.

## Data Availability

The data that support the findings of this study are available on request from the corresponding author.
